# Machine Learning and Meteorological Normalization for Assessment of Particulate Matter Changes during the COVID-19 Lockdown in Zagreb, Croatia †

**DOI:** 10.3390/ijerph19116937

**Published:** 2022-06-06

**Authors:** Mario Lovrić, Mario Antunović, Iva Šunić, Matej Vuković, Simonas Kecorius, Mark Kröll, Ivan Bešlić, Ranka Godec, Gordana Pehnec, Bernhard C. Geiger, Stuart K. Grange, Iva Šimić

**Affiliations:** 1 Know-Center, Inffeldgasse 13, 8010 Graz, Austria; mkroell@know-center.at (M.K.); bgeiger@know-center.at (B.C.G.); 2 Institute for Anthropological Research, Gajeva 32, 10000 Zagreb, Croatia; iva.sunic@inantro.hr; 3 Ascalia d.o.o., Ulica Trate 16, 40000 Čakovec, Croatia; mario.antunovic@ascalia.io; 4 Pro2Future GmbH, Inffeldgasse 25F, 8010 Graz, Austria; matej.vukovic@pro2future.at; 5 Institute of Epidemiology, Helmholtz Zentrum München, Ingolstädter Landstr. 1, 85764 Neuherberg, Germany; simonas.kecorius@helmholtz-muenchen.de; 6 Environmental Hygiene Unit, Institute for Medical Research and Occupational Health, Ksaverska cesta 2, 10000 Zagreb, Croatia; ibeslic@imi.hr (I.B.); rgodec@imi.hr (R.G.); gpehnec@imi.hr (G.P.); 7 Empa, Swiss Federal Laboratories for Materials Science and Technology, 8600 Dübendorf, Switzerland; Stuart.Grange@empa.ch; 8 Wolfson Atmospheric Chemistry Laboratories, Department of Chemistry, University of York, York YO10 5DD, UK

**Keywords:** random forests, LightGBM, air quality, coronavirus disease of 2019, PM_1_, PM_2.5_, PM_10_, traffic

## Abstract

In this paper, the authors investigated changes in mass concentrations of particulate matter (PM) during the Coronavirus Disease of 2019 (COVID-19) lockdown. Daily samples of PM_1_, PM_2.5_ and PM_10_ fractions were measured at an urban background sampling site in Zagreb, Croatia from 2009 to late 2020. For the purpose of meteorological normalization, the mass concentrations were fed alongside meteorological and temporal data to Random Forest (RF) and LightGBM (LGB) models tuned by Bayesian optimization. The models’ predictions were subsequently de-weathered by meteorological normalization using repeated random resampling of all predictive variables except the trend variable. Three pollution periods in 2020 were examined in detail: January and February, as pre-lockdown, the month of April as the lockdown period, as well as June and July as the “new normal”. An evaluation using normalized mass concentrations of particulate matter and Analysis of variance (ANOVA) was conducted. The results showed that no significant differences were observed for PM_1_, PM_2.5_ and PM_10_ in April 2020—compared to the same period in 2018 and 2019. No significant changes were observed for the “new normal” as well. The results thus indicate that a reduction in mobility during COVID-19 lockdown in Zagreb, Croatia, did not significantly affect particulate matter concentration in the long-term..

## 1. Introduction

Particulate matter (PM) is recognized as one of the major air pollutants affecting human health. Particle size plays an important role in determining pollutant respiratory deposition and thus potential health risks. Airborne particles PM_10_ (with aerodynamic diameter less than 10 μm) and especially its smaller fractions (e.g., PM_2.5_—with aerodynamic diameter less than 2.5 μm and PM_1_—with aerodynamic diameter less than 1 μm) are known to effectively enter the human body, e.g., trachea (upper throat) or bronchi, and even reach all the way down to the alveoli in the lungs, where it can penetrate from the lung alveoli into the blood [1,2]. In general, the smaller the particle size, the greater the adverse health effect [3,4,5]. Therefore, further reduction of PM pollution both in developed and developing countries has the potential to improve both life quality and expectancy. To better understand sources, as well as environmental and health impacts of air pollution, long-term measurement data sets are used in source appointment, epidemiological, and air quality studies [6]. On the other hand, short-term traffic bans can be used to pinpoint pollution contributors and raise awareness of air quality problems [7]. Ironically, besides causing worldwide health and economical disturbance, the current COVID-19 pandemic has also provided means to investigate air pollution [8]. Published evidence on the impact of the COVID-19 lockdown on the concentration of ambient air pollutants highlights the importance of transport and industrial activities [9,10]. For example, there is clear evidence for reduced gaseous (e.g., nitrogen dioxide (NO_2_)) and particulate pollutant concentrations in some urban areas, which can be linked to reduced transportation due to COVID-19 [11,12,13,14]. In contrast, the European Environment Agency reported that a consistent reduction of PM_2.5_ cannot be seen in European cities during the lockdown period [15]. The main reasons could be that local pollution sources are more various, including not only industrial activities and road traffic, but also the combustion of different fuels for the heating, as well as the formation of secondary aerosols [16]. Furthermore, it is not entirely clear how lockdown period pollutant concentrations depend on other effects/confounders that should be accounted for, e.g., weather effects [13]. The methods used in lockdown-related air pollution studies differ significantly. Many studies have tried to assess the influence of lockdown measures on air pollutant concentrations by a simple comparison of basic statistical parameters during the lockdown with the same period a year before or up to five years before [17,18,19,20,21,22,23]. However, in such studies, the variability of meteorological factors between years as well as long-term trends are not considered. There are not many studies that include more sophisticated statistical and modelling tools [13,24,25,26,27]. A summary of the review will be presented in the discussion section. In machine learning prediction of air pollutant concentration, one often assumes that the concentration (dependent variable) is a function of temporal and meteorological determinants (independent variables) [28,29,30]. With that in mind, one can employ complex algorithms which are considering possible non-linear relationships within the data and present true influencing determinates based on inference. Two commonly seen (non-linear) machine learning algorithms are Random Forest (RF) regression and neural networks (NN). In previous work [13] RF regression was used to predict pollutant concentrations during the lockdown in Graz and presented the advantages of utilizing such methods over the historical comparison of pollution. Similarly, RF was used to assess changes in pollutant levels during different stages of lockdown in Los Angeles by comparing predicted concentrations under different traffic emission scenarios [31]. A similar approach was used by Brancher [32] who refers to baseline models (non-lockdown periods) as “Business as usual” scenarios. The model describes hourly-averaged concentrations per pollutant and monitoring station to investigate air quality changes before and after lockdown and to verify the models’ predictive skill to reproduce the pollutant measurements. A NN approach was used to investigate whether changes in air quality in Nigeria were caused primarily by the lockdown. In this case, monthly average values of ground-level fine aerosol optical depth (AODf) across Nigeria from 2001 to 2020 were used [33]. Another method for the assessment of air pollution during the lockdown period is the difference-in-differences (DID) model. Xu et al. [34] used this method to evaluate air pollutants and air quality before and during the lockdown. The DID model calculates the effect of treatment (independent variable) on outcome (dependent variable) by comparing the average changes in each of the groups. In this case, the outcome is the level of air pollution. Control variables such as temperature, humidity, wind speed, etc. are also included. The model considers whether the lockdown was enforced or not for each date and based on this calculates relative changes in air pollution levels. The study by Gope et al. [35] used the Air Quality Index (AQI), which is calculated from the concentration of the pollutants, to analyse the impact of lockdown on the environment. Comparison of the AQI for these periods showed that most cities reduced their pollution. The pandemic of COVID-19 caused many changes in human activities, not only during the lockdown but also in the months following it. For example, working from home and virtual meetings caused lower mobility and, in some areas, even became a regular practice. There are mixed results published regarding the lockdown and “new normal” effects on particulate matter. While many methods are being used, there is a lack of a standardized approach for understanding these phenomena. In this work, the authors present an assessment of particulate matter in three mass fractions (PM_1_, PM_2.5_ and PM_10_) based on daily measurements over a long period of 12 years at one urban background location. Previous PM measurements at the same location have shown significant air pollution during winter months [36,37,38]. Data from air quality monitoring stations [39] shows that in a few years prior to the COVID-19 pandemic, levels of PM_10_ and PM_2.5_ were below regulatory limits set by Croatian and EU legislation for protection of human health. Annual averages of PM_10_ and PM_2.5_ were below 40 µg/m^3^ and 25 µg/m^3^, respectively. Daily limit value for PM_10_ is 50 µg/m^3^ and should not be exceeded more than 35 times during the calendar year. This criterion was met at that location since 2017. However, considering new WHO guidelines [40] which, in the light of recent scientific evidence, suggests much lower limit values for both, PM_10_ and PM_2.5_ fractions, it is necessary to apply new measures for reducing air pollution. With the aim to protect people’s wellbeing, it is important to know the main pollution sources and the efficiency of implemented measures. The intention of this paper was to examine whether a reduction in mobility during the COVID-19 lockdown caused changes in PM levels. The hypotheses are that the lockdown and the “new normal” both show reductions in particulate matter concentration. A reduction during the “new normal” is hypothesized due to a restriction on travel which affects Croatia’s tourism and more working from home. To test these hypotheses, these periods were evaluated by using ANOVA on the normalized (de-weathered) data. 

## 2. Materials and Methods

### 2.1. Particulate Matter and Meteorological Measurements 

Aerosol PM concentrations were measured in Zagreb, Croatia, at a sampling site located in the northern, residential part of the city (45°50′7″ N, 15°58′42″ E, 116 m a.s.l.,). 

**Figure 1:**
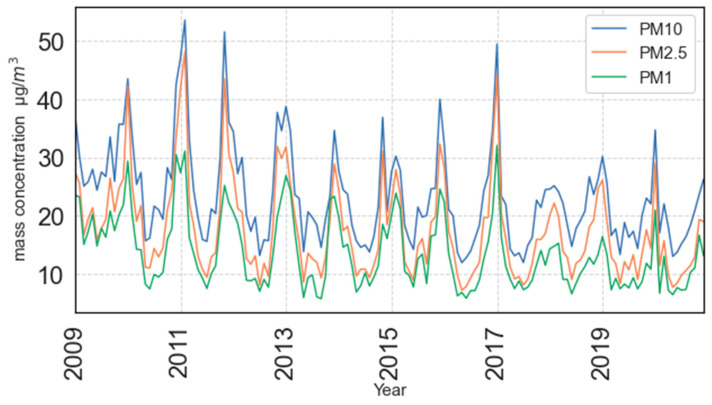
A time series plot of the collected particle mass concentration data from 2009–2020 for PM_1_, PM_2.5_ and PM_10_. For the sake of simplicity, the data is plotted as rolling monthly averages.

The area is characterized by modest traffic and population density. The household heating (gas and/or wood) season usually starts in October and lasts until April. The PM samplers (Sven Leckel, engineering office, Berlin, Germany) were positioned at about 20 m from the nearest street. Twenty-four-hour samples of PM_1_, PM_2.5_ and PM_10_ fractions have been collected continuously every day on quartz filters (47 mm in diameter) during 12-years period (2009–2020). PM mass concentrations were determined gravimetrically (Mettler TOLEDO MX5 balance, Greifensee, Switzerland) according to the EN 12341:1998 and EN 14907:2005 standards from 2009–2014 and EN 12341:2014 standard from 2015–2020. Before and after the sampling, filters were conditioned at a constant temperature (20 ± 1 °C) and relative air humidity (45–50% RH) for 48 h. Meteorological parameters (temperature, RH, wind speed and direction, pressure, and precipitation) were obtained from the Croatian Meteorological and Hydrological Service. For the input of data used in this study an explorative plot of the particle mass concentrations over time used in this study is shown in Figure 1. The collected data can be found at ref. [41].

### 2.2. Data Processing and Model Training

The dataset used in this analysis is air pollution data collected over the period of 12 years (2009–2020) in a daily frequency (daily average) in Zagreb, Croatia. The dataset contains the particulate matter mass concentrations of PM_1_, PM_2.5_ and PM_10_ and temporal information such as: day of week, Julian date (days counted from 1 January 1970), month, year, holiday tag, etc. In addition, temporally aligned meteorological influences were added into the dataset. These consist of maximum daily temperature (T), minimal daily T, difference of max and min T, average T, maximum and minimum daily pressure (p), difference of max and min p, average p, maximum daily relative humidity (RH), minimum daily RH, difference of max and min daily RH, average RH, wind speed and precipitation. Temporal and meteorological variables are given as independent or predictive variables. To retain a high amount of data for machine learning (ML), missing datapoints were imputed with backfill strategy (missing values are filled with the ones from the following day). Python programming language (www.python.org accessed on 1 February 2022, v3.7.10) was used for analysis, while data processing and model training follows the method and the process described in [13,30]. It is assumed that the concentrations of particulate matter (PM_1_, PM_2.5_, PM_10_; dependent variables) can be modelled through temporal and meteorological variables as independent ones, previously listed. To model the air pollution, RF [42] and LightGBM [43] methods were used. RF is an ensemble ML algorithm which consists of many individual decision trees and applies bootstrap aggregation (bagging) and feature randomness techniques in building each decision tree. Like RF, LightGBM is an ensemble method that relies on tree-based learning but utilizes gradient boosting techniques as well as different tree-building techniques. RF and LightGBM are non-parametric and as such require no formal distributional assumptions enabling these methods to deal with skewed and multi-modal data. Consequently, these methods are well suited for modelling challenging phenomena such as air pollution, but also other settings as outlined in several studies [13,28,30,44,45]. Air pollution ML models were trained for PM_1_, PM_2.5_ and PM_10_ respectively, with their daily concentrations representing target (dependent, predicted) variables. Following the method outlined in previous studies [46,47], hyperparameters of these regression models were optimized through 10-fold cross-validation with Bayesian optimization. The training dataset (TDS) consists of data between 1 January 2009 and 31 December 2019 while data from 2020 was split into several smaller datasets indicating different validation and interest periods. Finally, the models were tested on their generalization performance on MVS which is reported in the Results section. 

**Figure 2:**
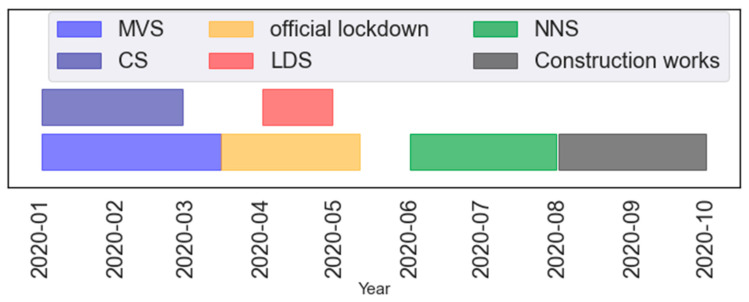
Datasets for 2020: model validation set-MVS (3 January–15 March); comparison set-CS (January and February); official lockdown (13 March–11 May); lockdown set- LDS (1–30 April); new normal set-NNS (1 June–31 July); construction works (March).

The first coronavirus patient was confirmed in Croatia on 25 February 2020. The Coronavirus Disease of 2019 (COVID-19) disease in Croatia was announced by the government on the 11 March 2020. In the second half of March, all public events and gatherings were canceled and all non-essential activities (shopping centers, bars and nightclubs, restaurants, cinemas, libraries, gyms, sports centers and sport events, dance schools, children’s workshops, religious and other public gatherings) were closed. On 19 March the decision was passed to restrict staying on the streets, squares, and other public places. Two days later it was followed by suspensions of public transport, suspension of intercity lines for trains and buses, and shortened working hours of shops and post offices. Finally, on 23 March a ban on leaving one’s place of residence or permanent residence was set and only persons and activities important for the movement and movement of goods were exempt. Relaxation of strict measures started on 23 April and was carried out in three phases till 11 May 2020: were relaxed between 27 April 27 and 11 May 2020 after which the bans were lifted. Sources regarding the given information can be found following references [48,49,50,51]. These interest periods and overall timeframes for 2020 are depicted in Figure 2 for the sake of simplifying the timeframes.

A priori, the 2020 data on “out-of-ordinary” events which might have affected the pollutant concentrations were analyzed. “Out-of-ordinary” events include: a 5.3-magnitude earthquake in Zagreb (22 March 2020), long-range transport of desert dust events on 26–30 March [13,52] and construction works near the measurement site in August 2020.

**Figure 3:**
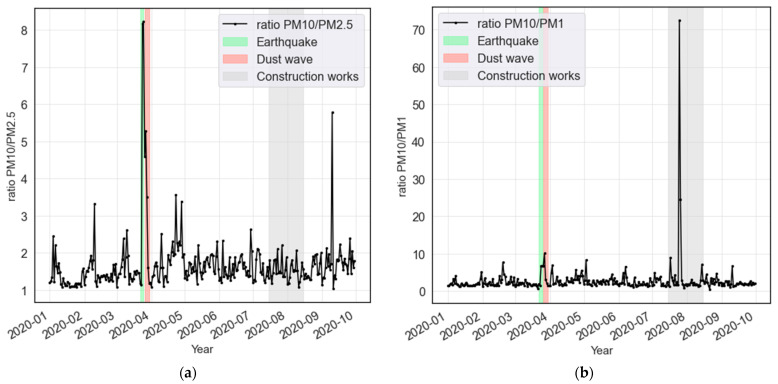
Out-of-ordinary events during 2020 shown as ratio between: (**a**) PM_10_/PM_2.5_ and (**b**) PM_10_/PM_1_. The events in March 2020 are assigned to either the Zagreb-earthquake on 22 March, the sand dust event between 24 and 30 March, or construction works at the site in early March, August, and September. These events were excluded from the analyzed timeframes.

As shown in Figure 3, these events clearly disrupt normal concentrations and introduce bias in the models. Therefore, the respective timeframes were excluded from the presented analyses. As a result, even though the lockdown timeframe lasted longer than the one given as LDS, several dust events needed to be excluded. A subset from MVS for comparison to LDS (comparison set or CS) was split, which is set between 3 January and 29 February 2020. The subset is shorter than MVS due to several construction activities at the site in March. LDS and NNS present the timeframe in focus of this lockdown pollution investigation. MVS was used to better understand the model generalization.

### 2.3. Meteorological Normalization (De-Weathering)

In this work, the authors followed the methodology from Grange et al. [28,29] for meteorological normalization of the daily particulate matter time series. Meteorological normalization was achieved by firstly creating an ML model per pollutant (particulate matter concentration) that generalizes well on unseen data. In the next step, all predictive variables (except Julian day) are repeatedly randomly sampled without replacement and used to predict pollutant concentration using the individual trained RF models. 

**Figure 4:**
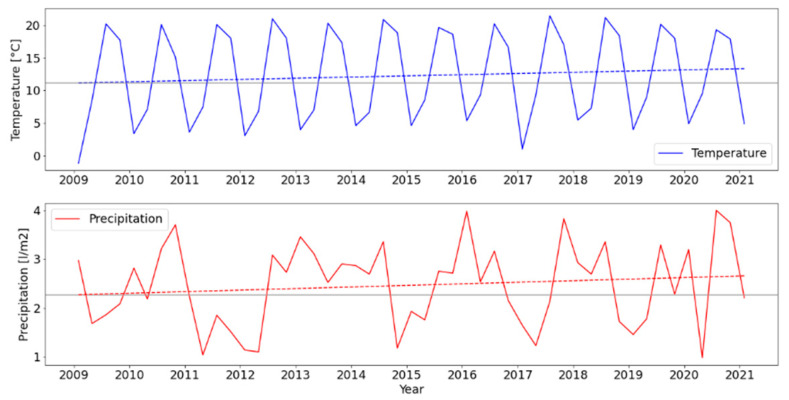
An overview of temperature and precipitation in Zagreb, Croatia through the studied timeframe 2009–2020. The data is plotted as a 3-month average and given trend-line by means of a regression line. The grey horizontal lines are the minimal values in of the regression line, showing the deviation from the minimal value.

The procedure of meteorological normalization removes the short-term variation in the time series. Reasoning for this procedure is that pollutant data must be corrected for meteorological and temporal effects which are changing over time and can therefore affect pollutant concentration. Herein, an example is shown by means of changing temperature and precipitation through the given years (Figure 4). One can observe that there was a trend in Zagreb, Croatia towards higher temperature and precipitation at the given monitoring station.

**Figure 5:**
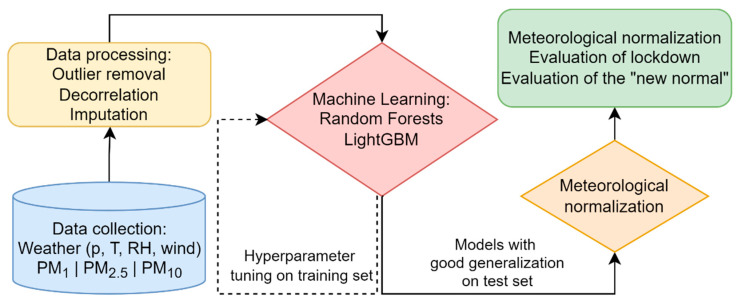
Schematics of the modelling procedure presented in this work.

To normalize, the model predictions for each pollutant for 100 random samples were then averaged into the normalized time series (normalized PM_1_, PM_2.5_, PM_10_). The complete procedure of data processing, machine learning model training and meteorological normalization is presented in Figure 5.

## 3. Results

The model scores by means of root-mean-square-error, *R*^2^ scores and chosen models by means of MVS performance are shown in Table 1. Based on *R*^2^ scores, all three models show good predictive values (good generalization). The prediction quality in RMSE is similar for PM_10_ and PM_2.5_, while it increases for PM_1_. When comparing the *R*^2^ scores to the author’s previous work [13], the observed values in this study suggest a reasonably good generalization with *R*^2^ scores above 0.77.

**Figure 6:**
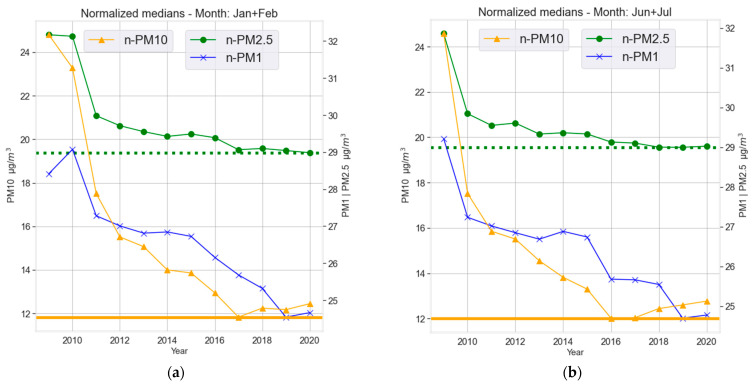
(**a**) Normalized medians for January and February (CS) during 2009–2020, (**b**) Normalized medians for June and July (NNS) during 2009–2020. The green line is PM_10_ related to the respective right axis, while blue and orange are PM_1_ and PM_2.5_ mass concentrations respectively referred to the left axis. The horizontal lines present the lowest values for the axes respectively.

**Figure 7:**
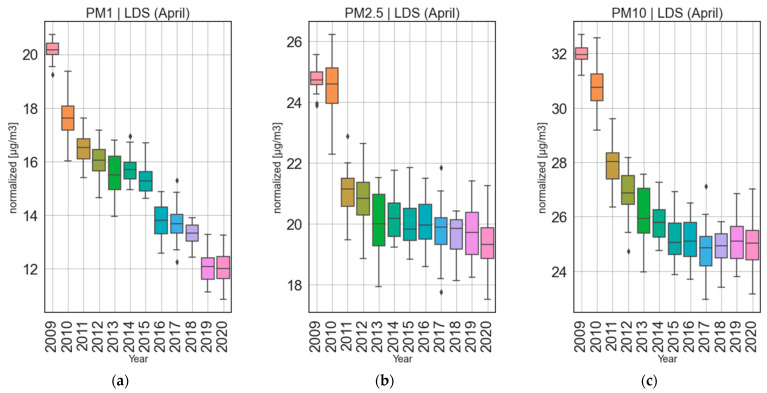
Boxplots of April normalized concentrations through the years for (**a**) PM_1_, (**b**) PM_2.5_, (**c**) PM_10_. The boxes show the quartiles of the dataset while the whiskers show the rest of the distribution, except for points that are determined to be “outliers” (diamond-shaped markers) by being outside of the inter-quartile range.

Once models were trained, the data was normalized (de-weathered) as described in Section 2.3 and depicted in Figure 5. To evaluate change in airborne pollution concentrations due to the lockdown, yearly trends were assessed by means of median of the normalized time series. Three timeframes were compared (Figure 6), namely the months of January and February together (CS) and June together with July (NNS) which are considered to be the new normal (post-lockdown changes). Normalized time series during the months of April (LDS) every year is given in Figure 6. In Figure 6a, for CS (January and February, pre-lockdown reference) there is a continuous reduction from 2009 to 2017 for all size fractions of PM. However, starting with 2017 an unexpected increase for the normalized values can be observed for PM_10_ and a slight increase from 2019 to 2020 for PM_1_, while PM_2.5_ has a steady level from 2017 onwards. During NNS (the months of June and July, Figure 6b) a reduction in pollution compared to previous years was expected due to lower tourism rate and the travel ban as well as working from home and many isolations. Instead, the observed pattern for NNS is like CS, with PM_2.5_ showing similar levels from 2018–2020, while PM_1_ and PM_10_ show slight increases. In the case of April, when the lockdown took place (LDS; boxplots in Figure 7), PM_2.5_ shows a steady decrease from 2009 onwards with a visually insignificant decrease from 2019 to 2020. PM_10_ mass concentration shows a similar trend with visually insignificant changes from 2017 onwards. The results are slightly different from PM_1_ which shows mixed periods of decrease and increases from 2009 onwards. Even though there seems to be a huge drop from 2018 to 2019, there is an increase from 2019 to 2020. ANOVA was utilized to assess whether the changes observed in normalized PM-time series are significant. 

For each of PM_1_, PM_2.5_ and PM_10_ a test was created with the year as categorical variable (2018, 2019, 2020) as the independent variable and the normalized concentrations as the dependent variable. ANOVA showed that yearly changes for April do not show statistical significance for any of the PM fractions—PM_1_ (*p* = 0.10), PM_2.5_ (*p* = 0.47) and PM_10_ (*p* = 0.76). Similar results were obtained for the NNS (new normal) with PM_1_ (*p* = 0.26), PM_2.5_ (*p* = 0.81) and PM_10_ (*p* = 0.72).

## 4. Discussion

Table 2 presents the literature findings on the influence of lockdown on air pollutant’s levels and methods applied. It is evident that most of the studies did not include long-term PM measurements and were mostly focused on the comparison of the lockdown period with the same period in 2019. Furthermore, they do not account for the effect of year-to year variability and seasonal variability caused by meteorology and weather conditions, which was the reason for using normalization [28,29]. However, even normalization can suffer from a lack of data or model quality. For this reason, we utilized, besides RF, also LightGBM and Bayesian optimization for tuning all models to improve model accuracy. Comparing to data sources listed in Table 2, this work has a fair amount of data with a collection starting from 2009, which the authors deem to be needed for such an undertaking. 

Regarding the changes in air pollution, the results obtained in this study are in line with the other published work [13,14,34,73]. In a previous study carried out in Zagreb, average concentrations of NO_2_, PM_1_ and PAHs in PM_1_ during the lockdown period were compared with the average concentrations for the same period in 2019. It was found that during lockdown at the traffic measuring site concentrations decreased by 35% for NO_2_ and PM_1_ compared with the same period in 2019. However, at the urban background measuring site NO_2_ decreased by 27% while PM_1_ levels remained like the year before [18].

There is mixed evidence for whether PM mass concentrations were truly affected by the lockdown, i.e., change in mobility, at least for the sites not heavily affected by traffic. In the authors previous work [13], it was found that a drop in PM10 mass concentration during the lockdown in Graz, Austria is rather inconsistent when comparing it to gaseous pollutants (e.g., NO2). A drop in NO2 gas concentrations was found to be around 40%, while PM_10_ mass concentration decrease was in the range of 6–14% compared to previous years. However, these analyses were conducted without meteorological normalization. Given the comparison to the observed reduction in traffic, it can be assumed that the reduced traffic was not a dominant contributing factor to the changes in PM_10_ atmospheric load. Xu et al. [34] who were using the difference-in-differences method to compare air pollution before and during the lockdown in China, found no change in PM_10_ and PM_2.5_ concentrations. This finding implies that traffic cannot be considered the main PM source. Although particulate matter concentrations show short-term declines within three days after the lockdown policies were implemented, when compared to the measurements from different monitoring sites that acted as a control group, the lockdown effects were not as pronounced. The given results are in accordance also with a study by Etchie et al. [33] who observes no effect on PM by the lockdown. A study that also used RF and meteorological normalization shows only a moderate decrease for PM_10_ [73]. The results imply that in Zagreb, Croatia, traffic is not the main contributor in such a site to air pollution by means of particulate matter. This is essential to the discussion of which factors contribute to public health, such as traffic, residential heating, and urban planning. If traffic is not the main contributor to particulate matter, regardless of its contribution by means of nitrogenous oxides [13] and polycyclic aromatic hydrocarbon (PAH) [18] then local policymakers need to invest more efforts in understanding other contributors and prevention policies which will improve air quality.

### Limitations and Future Work

First and foremost, there are many contributing factors to atmospheric PM levels, such as secondary particle formation and long-range transport of particulate matter which are here not considered. Beyond those, there are also solar irradiation, traffic density, contribution from resuspension of road dust, etc. 

The height of the boundary layer changes significantly during the day. In summer, this is more pronounced because the sun warms the substrate, vertical currents appear, and the height of the boundary layer increases. As night approaches, the height of the boundary layer decreases. Since in this work the authors used the daily values of the concentrations of suspended particles, this daily course is not distinguishable in the given data on the concentration of PM. Furthermore, in the city of Zagreb, a high stability of the boundary layer of the atmosphere was observed during the cold part of the years when increased concentrations of PM were also observed. It should be emphasized that Zagreb is surrounded by Medvednica mountain on the north and the river Sava on the south, and there are no major industrial cities along these routes. East and west of Zagreb there are only suburban settlements (Zaprešić, Samobor, Sesvete, Dugo Selo) which are residential centers without pronounced dominant sources, and this contribution is not included in this paper.

Another limiting factor is also the data frequency and measurement method. This work is based on daily averages of particulate matter and meteorology which are rough estimates of pollution trends and their dependence on meteorology. Even though the models have good generalization with *R*^2^ scores above 0.77, using these predictions will lead to error propagation since the models do not cover all the variance. 

In the future, the authors aim to shift the focus towards PM composition and source apportionment regarding changes during the lockdown which helps understand the contributions. Furthermore, the authors intend to apply recent in-house research on the intersection of physics-based and machine learning based models (so call physics-inspired machine learning) such as seen in recent research [45,74] where models’ accuracy profits from the combination of the two worlds.

## 5. Conclusions

In this work the effects of the lockdown on PM mass concentration in Zagreb, Croatia were evaluated. The authors hypothesized that the COVID-19 lockdown (April 2020) and the “new normal” (June, July 2020) would both exhibit a decrease in PM_1_, PM_2.5_, and PM_10_ mass concentrations due to changed human behavior and mobility. To investigate the anticipated decrease in PM mass concentrations, machine learning, by means of Random Forests (RF) and LightGBM (LGB) were utilized and combined with meteorological normalization. The RF and LGB models trained in this study exhibited a reasonably good generalization on the test set (*R*^2^ scores > 0.77). By using normalization, the trend component of the PM mass concentration was extracted and compared it pre-, during, and post-lockdown timeframes. The results by means of normalized concentrations show that over the course of 2009–2017/2018, for the city of Zagreb at an urban location, PM mass concentrations dropped, however, no significant changes were observed in PM mass concentrations due to the lockdown or post-lockdown events. Besides that, given that NO_2_ reductions were earlier observed at the same site, one can speculate that a reduction in mobility did not affect particulate matter to a significant extent at this specific site.

## Figures and Tables

**Table 1:** Results of the machine learning models for PM-concentrations shown on the validation set (MVS).

Pollutant	RMSE	*R*^2^ Score	Winning Algorithm
PM_10_	10.47	0.77	Random Forests
PM_2.5_	9.87	0.78	Random Forests
PM_1_	6.49	0.77	LightGBM

**Table 2:** Literature findings on the lockdown’s effects on particulate matter concentration. Abbreviations used: Machine learning (ML), Descriptive statistics (DS), Modelling (MD), Unsupervised methods (UM), Meteorology (Met), Machine learning with normalization (MLN).

Geographic Location	Pollutants	Methods	Data Used	Ref.
Zagreb, Croatia	**PM_10_**, **PM_2.5_**, **PM_1.0_**	MLN	Training: from 1 January 2019 to 31 December 2019 (114 samples) Validation: 3 smaller datasets in 2020 (10 samples); Test: 4 May to 13 May 12020 (10 samples)	This study
Zagreb, Croatia	NO_2_, **PM_10_**	DS	Comparison between lockdown period (26 February–7 May 2020) and the same period in 2019	[17]
Zagreb, Croatia	NO_2_, **PM_1.0_**, PAHs	DS	Comparison between lockdown period (March–May 2020) and the same period in 2019	[18]
Novi Sad, Serbia	**PM_2.5_**, NO_2_, NO, NO_x_, CO, SO_2_ + Met	DS	Comparison before and after entering the state of emergency (1 February to 30 April)	[53]
Skopje, Bitola, Tetovo, Kumanovo, Macedonia	**PM_10_**, **PM_2.5_**, NO_2_, O_3_, CO, Met	DS	Comparison of COVID19 period (last week of February 2020 to the end of May 2020) with the same period in 2017–2019 (nonCOVID-19 period)	[19]
Milan, Italy	**PM_10_**, **PM_2.5_**, O_3_, NO_2_, SO_2_, CO, air quality index (AQI) + Met	DS	Comparison between pre-lockdown (January–February 2020) and lockdown period (March–April 2020)	[54]
Milan, Italy	**PM_10_**, **PM_2.5_**, BC, benzene, CO, NO_2_, O_3_, NO_x_ + Met	DS	Comparison between periods: CTRL (from 7 February 2020 to February 20), PL (from 9 March 2020 to 22 March 2020), and TL (from 23 March 2020 to 5 April 2020)	[55]
Milan, Bologna, Florence, Rome, Naples, and Palermo, Italy	**PM_10_**, **PM_2.5_**, NO_2_, O_3_ + Met	DS	Comparison between 2019-period (25 February–2 May 2019) and 2020-period (24 February–30 April 2020)	[20]
Athens, Greece	**PM_2.5_**, **PM_1.0_**, eBC, EC, OC, paricle number size distribution, SO_4_^2^-, NO^3-^, Cl^-^, NH^+^ + Met	DS	Comparison of reference period (1 January–10 March 2020) the two lockdown periods (11 March–22 March 2020 & 23 March–12 April 2020) with the respective periods in 2018 and 2019	[56]
Barcelona & Catalonia, Spain	NO_2_, O_3_, **PM_10_**—hourly samples	DS	Comparisons during the before (15 February to 13 March), during (14 March to 21 June) and after lockdown (22 June to 31 August)	[57]
Barcelona, Spain	**PM_10_**, NO_2_, SO_2_, O_3_, BC + Met	DS	Comparison for the periods before (16 February to 13 March) and during the lockdown (14 March to 30 March)	[58]
Madrid, Barcelona, Spain	NO_2_—hourly samples + Met	DS	Comparison of March in the years 2018, 2019 and 2020	[21]
South East of the UK	NO_2_, **PM_2.5_**, **PM_10_**, O_3_ + Met	DS	Comparison between lockdown period (March–May 2020) with the same period in 2015–2019	[22]
UK	NO, NO_2_, NO_x_, O_3_, **PM_10_**, **PM_2.5_**—hourly samples	DS	Comparison between lockdown period (1 January to 30 June 2020) with the period from 1 January 2015 to 31 December 2019	[23]
London, Glasgow, Belfast, Birmingham, Manchester and Liverpool, UK	NO_x_, SO_2_, **PM_2.5_**, O_3_ + Met	DS	Comparison of 100 days post-lockdown (23 March to 30 June 2020) with the same period from the previous 7 years	[59]
Turkey	**PM_10_**, SO_2_,	DS	Comparison of 2020 to the average of the 5-year period (2015–2019)	[60]
Baghdad, Iraq	NO_2_, O_3_, **PM_2.5_**, **PM_10_**_,_ AQI	DS	Comparison of the periods before the lockdown from 16 January to 29 February 2020, and during four periods of partial and total lockdown from (1 March to 24 July 2020)	[61]
Kuwait	**PM_10_**, **PM_2.5_** + Met	DS	Comparison between the lockdown in 2020 with the corresponding periods of the years 2017–2019	[62]
India	**PM_2.5_**, **PM_10_**, NO_2_, O_3_, CO, SO_2_ + Met—hourly	DS	Comparison between lockdown period (25 March–3 May 2020) and the same period in 2017–2019	[63]
Southern regions of India	**PM_2.5_**, **PM_10_**, NO, CO, O_3_	DS	Comparison between lockdown period (1 April to 31 July 2020) and the same periods in 2018 and 2019	[64]
Kolkata City, India	**PM_10_**, **PM_2.5_**, O_3_, SO_2_, NO_2_, CO	UM	Comparison of lockdown period (25 March to 15 May 2020), with the similar time frame in 2017, 2018 and 2019	[24]
Sao Paulo, Brazil	NO, NO_2_, CO, **PM_2.5_**, **PM_10_**, SO_2_, O_3_, NO_x_	DS	Comparison the partial lockdown periods (25 February 2020 to 23 March 2020 and from 24 March 2020 to 20 April 2020) to the five-year monthly trend (February, March and April of the years 2015, 2016, 2017, 2018 and 2019)	[65]
Nice (France), Rome and Turin (Italy), Valencia (Spain) and Wuhan (China)	NOx, **PM_2.5_**, **PM_10_**, O_3_	DS	Comparison of lock down period (1 January 2017 until 18 April 2020) with the same period over the three previous years (2017–2019)	[66]
sixteen selected cities located in South Asia, East Asia, Europe, and North America	NO_2_, CO, **PM_2.5_**, O_3_, SO_2_	DS	Comparison between from 1 January–15 May for the year of 2015–2019 (defined as baseline period) and 2020 (lockdown)	[67]
50 most polluted capital cities in the world	**PM_2.5_**, AQI	DS	Comparison between before and during quarantine	[68]
34 countries	NO_2_, O_3_, **PM_2.5_**	DS	Comparison between from 1 January–15 May for the year of 2017–2019 and 2020 (lockdown)	[69]
Multiple locations *	NO_2_, SO_2_, CO, O_3_, **PM_10_**, **PM_2.5_**, AQI	DS	Comparison between lockdown period in 2020 to the same period of 2017, 2018 and 2019	[70]
New York, Los Angeles, Zaragoza, Rome, Dubai, Delhi, Mumbai, Beijing and Shanghai	**PM_2.5_**	DS	Comparison of lockdown period (December 2019–March 2020), and the same period in earlier years 2017–2019	[71]
São Paulo in Brazil; Paris in France; and Los Angeles and New York in the USA	NO_2_, CO, **PM_2.5_**, O_3_ + meteorology	DS	Comparison of March in the years 2015–2020	[72]
Graz, Austria	NO_2_, **PM_10_**, O_3_, O_x_ + Met	ML	Training: from 3 January 2014 to 31 December 2019 (daily) Validation: from 3 January 2020 to 10 March 2020 (daily), Test:l lockdown set, LD (10 March 2020 to 2 May 2020—daily samples), and a hard lockdown set, HLD (20 March 2020 to 14 April 2020—daily samples)	[13]
Lombardy, Italy	NO_2_, **PM_2.5_** + Met	ML	Training: from 2012 through 2019 Validation: months from January to April for 2016–2020 Test: from January through early May 2020	[25]
Sao Paulo, Brazil	CO, O_3_, NO_2_, NO, **PM_2.5_**, **PM_10_** + Met	ML	Training: from 1 January to 23 April 2020 (114 samples); Validation: 24 April to 3 May 2020 (10 samples); Test: 4 May to 13 May 2020 (10 samples)	[26]
Quito, Ecuador	CO, NO_2_, **PM_2.5_**, SO_2_, O_3_	MLN	Training: from 1 January 2016 to 15 January 2020 (2 months before the COVID-19 lockdown) Test: from 16 January 2020 to 15 March 2020 (the day of the national lockdown).	[27]
Cantabria, Spain	NO_,_ NO_2_, **PM_10_**, O_3_, Met	MLN	Data from 11 stations (2013–2020) Training data 2013–2019, test set lockdown and new normal 2020	[73]
Vienna, Austria				

* Wuhan, Beijing (China), Delhi (India), Tehran (Iran), Istanbul (Turkey) in Asia; Rome (Italy), Madrid (Spain), Paris (France), London (UK), Berlin (Germany) and Moscow (Russia) in Europe; Johannesburg (South Africa) in Africa and Los Angeles, New York City (USA), Mexico city (Mexico), Sao Paulo (Brazil) and Lima (Peru) in North and South America.

## Data Availability

Data is available at [41].
